# New mutants defective in RMED/V neuron specification are alleles of EOR-1 and EOR-2

**DOI:** 10.17912/micropub.biology.000139

**Published:** 2019-07-31

**Authors:** Xun Huang, Yishi Jin

**Affiliations:** 1 MCD biology, University of California, Santa Cruz, CA95064; 2 Institute of Genetics and Developmental Biology, Chinese Academy of Sciences, Beijing, 100101, China; 3 Neurobiology Section, Division of Biological Sciences, University of California, San Diego, CA92093

**Figure 1:**
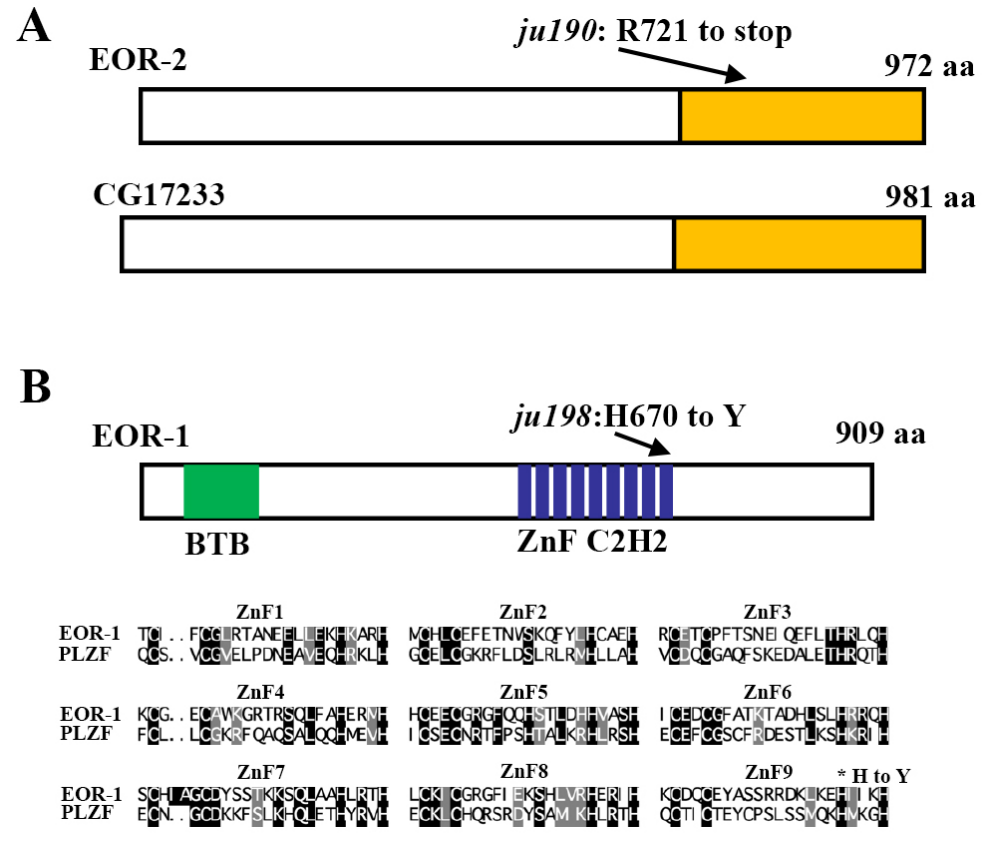
EOR-1 and EOR-2 are required in RMED/V neuron specification.(A) EOR-2 is a novel protein with moderate homology in the C-terminal (boxed) to *Drosophila* protein CG17233. *ju190* is a stop codon mutation. (B) EOR-1 contains BTB domain and nine C2H2 Zing fingers (ZnF). Alignment of nine zinc finger domains from EOR-1 and PLZF is shown. *ju198* changes the histidine (H) in last zing finger to tyrosine (Y).

## Description

In a genetic screen for genes affecting RMED/V neuron specification, we isolated two mutants, *ju190* and *ju198* (Huang et al., 2002; Huang et al., 2004; Huang and Jin, 2019). We mapped *ju190* to the X chromosome, a region covered by three cosmids (H01A20, C44H4 and F54E4), between *unc-9* and *unc-3*, using the snip-SNP mapping strategy. The novel conserved nuclear transcription factor *eor-2* is contained in the cosmid C44H4, and *eor-2(cs42)* mutant animals exhibit similar behavior defects as *ju190* (Rocheleau *et al.*, 2002). We introduced P*_unc-25_*GFP into *eor-2*(*cs42),* a null allele, and found no expression in RMED/V cell, as for *ju190* (Huang and Jin, 2019). DNA sequencing analysis of the *eor-2* genomic DNA from homozygous *ju190* animals identified a C to T nucleotide transition that results in an Opal stop at Arg721, in the conserved C-terminal domain (Figure 1A). Therefore, the RMED/V defects in *ju190* arise from a complete loss of EOR-2 function.

We mapped *ju198* to chromosome IV in a region near *eor-1*. EOR-1 is a functional binding partner of EOR-2 (Howard and Sundaram, 2002; Howell *et al.*, 2010). The phenotypic similarities between *eor-1* and *eor-2* and between *ju198* and *ju190* led us to suspect that *ju198* might be an allele of *eor-1*. Indeed, we found that *eor-1(cs28),* a null allele, failed to complement *ju198* for the RMED/V phenotypes (Huang and Jin, 2019). *eor-1* encodes a *C. elegans* ortholog of mammalian promyelocytic leukemia zinc finger protein (PLZF) with a BTB domain and nine C2H2 zinc fingers (Figure 1B) (Rocheleau *et al.*, 2002). We found that *ju198* is a missense mutation changing a conserved histidine to tyrosine in the last zinc finger (Figure 1B). Altogether, our data show that the complete loss of either *eor-1* or *eor-2* function results in identical differentiation defects in RMED/V neurons.

## Reagents

Strains are: CZ2014 *eor-1(ju198), juIs76*; CZ2006 *eor-2(ju190); juIs76*
